# Highly Educated Immigrant Workers’ Perspectives of Occupational Health and Safety and Work Conditions That Challenge Work Safety

**DOI:** 10.3390/ijerph19148757

**Published:** 2022-07-19

**Authors:** Janki Shankar, Daniel Lai, Shu-Ping Chen, Tanvir C. Turin, Shawn Joseph, Ellen Mi

**Affiliations:** 1 Faculty of Social Work, University of Calgary, Calgary, AB T2N 1N4, Canada; shawn.joseph@ucalgary.ca (S.J.); yilin.mi@ucalgary.ca (E.M.); 2 Faculty of Social Sciences, Department of Social Work, Hong Kong Baptist University, Hong Kong, China; daniel_lai@hkbu.edu.hk; 3 Department of Occupational Therapy, University of Alberta, Edmonton, AB T6G 2R3, Canada; shuping2@ualberta.ca; 4 Department of Family Medicine, Cumming School of Medicine, University of Calgary, Calgary, AB T2N 1N4, Canada; turin.chowdhury@ucalgary.ca; 5 Department of Community Health Sciences, Cumming School of Medicine, University of Calgary, Calgary, AB T2N 1N4, Canada

**Keywords:** new immigrant workers, highly qualified, occupational health and safety, workplace challenges, policy, practice

## Abstract

This study explored the perspectives of new immigrant workers regarding occupational health and safety and workplace conditions that increase workers’ vulnerability to sustaining injury or illness. Using an interpretive research approach and semi-structured qualitative interviews, 42 new immigrant workers from a range of industries operating in two cities in a province in Canada were interviewed. Seventy-nine percent of the workers were highly qualified. A constant comparative approach was used to identify key themes across the workers’ experiences. The findings revealed that new immigrant workers have an incomplete understanding of occupational health and safety. In many workplaces, poor job training, little worker support, lack of power in the workplace, and a poor workplace safety culture make it difficult for workers to acquire occupational health and safety information and to implement safe work practices. This study proposes workplace policies and practices that will improve worker occupational health and safety awareness and make workplaces safer for new immigrant workers.

## 1. Introduction

International labor migration is an important part of global social and economic development and the United Nations 2030 Sustainable Development Agenda recognizes migration as an important aspect in development policy [1]. In 2019, the United Nations estimated the stock of international working-age immigrants (aged 15 and over) worldwide to be 245 million, with nearly half of all immigrants working in either North America or in Northern, Southern, or Western Europe [2]. As regards their origin, one-third of the international immigrants originate from the Asia and Pacific region, followed by Europe and Central Asia, the Americas, Africa and the Arab States [2]. Immigrants make important contributions to the economic prosperity of their destination countries. The declining fertility and the decrease in working-age populations have led to a rising demand for immigrant workers to sustain the national economies of many developed countries. The remittances sent by immigrants to their families back home are a major source of capital in developing countries, helping to reduce poverty and stimulate economic development [2]. Despite their contributions to the world economy, immigrant workers, especially new immigrants (those in the destination country for less than 10 years) [3] who are ethnic and racial minorities without local language proficiency, are among the most vulnerable members of society [4,5,6]. These workers are often engaged in unskilled and “precarious employment” (also called 3D jobs—Dirty, Demeaning, and Dangerous) [4], characterized by high insecurity, unsafe work, long work hours, high workload, low wages, and stressful psychosocial working conditions that include racism and xenophobia, especially where migration is perceived to take away jobs from native workers [4,5,7,8]. Worldwide, immigrants engaged in precarious employment take greater risks on the job compared to native-born workers, do work without adequate training or protective equipment, and do not complain about unsafe working conditions. They have higher rates of workplace injury, occupational fatality, and poor health outcome compared to native-born workers [4,9]. The higher rates of occupational injury and fatality among these workers can be attributed to a variety of factors; these include the precarious nature of the jobs immigrants take on and the lack of occupational health and safety (OHS) training these immigrant workers receive [4].

Labor migration and exploitation of ethnic and racialized immigrants from developing countries is not a new phenomenon. It has been in existence since the birth of modern capitalism in the Global North in the early nineteenth century and its worldwide spread by the end of the twentieth century [6,10]. Central to the formation of global capitalism was the creation of a world market in labor and, historically, this was a key function of Western colonialism and imperialism. With the rise of new-world capitalism, controlled by transnational corporations in the latter decades of the twentieth century, there were massive new migrations worldwide. These created a new global working class, comprising a significant proportion of ethnic and racialized immigrants that labor in factories, on farms, commercial establishments, and offices for the global economy [6,10]. The twenty-first century ushered in a crisis of global capitalism fueled by free-trade agreements and neoliberal policies of nation states [6]. This has generated a surplus of under- and unemployed immigrant labor in the global labor market. As a result, the transnational capitalist class has been able to forge a new capital–labor relationship based on these flexible labor arrangements, under which workers can be hired and fired at will and enjoy no stability [10,11]. While many of these workers might have status as legal immigrants, those who are undocumented workers (lack legal authorization to work) risk extreme forms of exploitation by unscrupulous employers [4,6,10]. The current research is about new legal immigrants (those who have legally migrated to Canada less than ten years ago for work purposes) [3].

Canada has one of the highest rates of immigration among the Organization for Economic Co-operation and Development (OECD) countries, and the lowest disparity in labor force participation between Canadian-born and immigrant employees [12]. Nevertheless, new immigrants face several challenges finding employment that are commensurate with their relatively high level of educational qualifications and experience; this is particularly the case for linguistic (who speak only non-official languages at home) and visible minorities (persons, other than indigenous people, who are non-Caucasian in race or non-white in color) [13]. Because of language barriers and the time required for overseas credentials to be recognized by Canadian regulatory bodies, many of these immigrants take up precarious employment. In Canada, precarious employment is prevalent among new immigrants, especially visible and linguistic minorities, making them more vulnerable to occupational injury and illness [14,15]. They become vulnerable when they enter the Canadian labor market with limited knowledge of OHS and work-related hazards [16,17]. While having a good understanding of OHS can be empowering for all workers, it is crucial for these new immigrant workers who experience additional challenges with respect to language, class, race, and social conventions [18].

Immigrants need to learn safe work practices; they need to have the confidence to refuse unsafe work, and they need to know how to access compensation for workplace injuries [19]. A poor understanding of workplace hazards and fears of losing their job and causing trouble often induce immigrant workers to take on unsafe work or fail to pursue compensation for an injury [20]. The situation is most critical for undocumented immigrant workers who also risk being deported to their country of origin [9]. An immigrant worker who is not apprised of OHS practices might experience financial and mental distress after claiming injury compensation from an organization, such as the Workers’ Compensation Board (WCB) [21]. While existing studies have contributed much to knowledge about new immigrant workers’ high vulnerability to occupational injury, there is relatively little information about immigrant workers’ perspectives on OHS and the challenges they experience to practice workplace safety. Such information could be used to design programs and interventions that address the risks that new immigrants face in the workplace and the costs associated with workplace injuries when they occur. The current study, conducted in the province of Alberta in Canada, examines these issues and provides suggestions for their rectification.

### 1.1. OHS and Immigrant Workers

The goals of Occupational Health and Safety (OHS) are to inform workers about workplace hazards. OHS standards in Canada require that the physical and mental health of the worker are considered when work is assigned and that the working conditions promote worker safety [22]. The OHS concept includes the recognition of a worker′s right to economic and medical support in a case of work injury; such support is referred to as workers′ compensation or social insurance [8]. Smith and colleagues [22] developed an OHS vulnerability framework and a 29-item questionnaire designed to measure workers′ vulnerability to occupational health and safety (OHS) risks. The framework identifies four work-related dimensions that increase the risk of injury for all workers: exposure to workplace hazards, inadequate workplace protections and policies, low worker awareness of occupational health and safety, and a culture that discourages worker participation in safety practices. These four dimensions can be applied to new immigrant workers because they are likely to engage in precarious employment with little or no training, and they have limited understanding of occupational health and safety standards and workplace rights. Immigrant workers are more likely than native-born workers to perform physically demanding jobs [23,24,25,26], to be exposed to OHS hazards [14,27], and to lack health and safety insurance [28]. Immigrant workers are also less likely than native-born workers to receive formal job training and information regarding onsite health and safety practices [14]. Many new immigrant workers do not feel empowered to voice their health and safety concerns, to ask questions about health and safety, and to refuse unsafe duties [9]. Therefore, immigrant workers experience higher rates of work-related injuries and illnesses than native-born workers [4].

### 1.2. OHS Legislation in Alberta

Alberta receives the fourth largest share (after Ontario, British Columbia, and Quebec) of immigrants to Canada [29]. Occupational health and safety legislation in Alberta seeks to protect workers from worksite-related hazards. Alberta′s injury prevention laws are premised on workplace safety being a shared responsibility of employers and workers. Alberta has a high level of workplace injury. A comparison of the 2015–2017 average rates to the 2018 rates showed that among provinces with over 100,000 employees, Ontario and New Brunswick showed the greatest increase (15%) in lost-time injury rate, followed by Alberta (13%) [30]. The Alberta OHS Act outlines the general rights and responsibilities of employers and workers [31]. In 2017, the Alberta Government undertook a comprehensive review of OHS legislation enacted in 1976 [32]. The OHS Act gives workers the right to know about hazards in the workplace, the right to participate in hazard identification and remediation, and the right to refuse work that might affect their health and safety or the health and safety of others. Workers are responsible for working safely, for asking for training if they do not know how to do a job, and for reporting unsafe work practices to their employer. Workers also must refrain from harassment or violence in the workplace and must use the safety equipment and clothing required for the job they are performing [33].

Under the OHS Alberta Act, employers must protect the health, the safety, and the welfare of their workers. These responsibilities include ensuring that workers have the skills and training to do their jobs safely and ensuring that safety data are readily available to workers. OHS legislation cites the following: workers must be aware of their rights and responsibilities, workers must be aware of workplace health and safety issues, workers must be protected from harassment or violence at the work site, and workers must be supervised by supervisors who are competent and who are familiar with relevant OHS legislation [33]. Employers convicted of offenses with respect to OHS legislation can be penalized by fines, imprisonment, corporate probation, or a combination of these penalties [34]. The Workers Compensation Board (WCB) in Alberta provides rehabilitation services and wage-loss support for workers with job-related injuries and illnesses. The WCB works with Alberta OHS to reduce the impact of workplace injuries and illnesses on Albertans [35].

An increasing body of research highlights that new immigrant workers have a poor understanding of OHS. Information regarding immigrant workers’ understanding and practice of OHS in the Canadian context is scarce. The current study uses a qualitative design to explore these issues. Two key areas investigated were: (1) What do new immigrant workers understand by OHS in Canada? (2) What workplace conditions limit the ability of new immigrant workers to work safely?

Research questions: (1) How do recent immigrant employees perceive occupational health and safety? (2) What work conditions pose challenges to the safety of recent immigrant employees?

## 2. Materials and Methods

### 2.1. Research Design and Data Collection

This study adopted an interpretative research approach to explore the research questions [36]. The research investigators and the graduate research assistants who comprised the research team are themselves immigrants and were drawn from occupational therapy and social work backgrounds. Their immigrant experiences played an important part in the understanding of participants′ challenges and in the co-creation of data [36]. Semi-structured interviews were used to examine: the participant′s understanding and perspective of OHS, the safety training received by the participant, the job hazards the participant observed and experienced, the participant′s knowledge of post-injury worker support services, and the physical and emotional challenges the participant faced in his/her work environment. Since all the participants reported that they could understand and speak English, the interviews were conducted in English. However, since several participants had language limitations and insufficient vocabulary to describe their experiences, the researchers used some strategies to address these issues. They gave participants ample time to complete what they wanted to convey and used simple sentences and examples to explain a question or concept. The researchers also frequently used paraphrasing to ensure that they had accurately captured what the participant said. The researchers were aware of and attentive to the power differential between themselves as graduate academic researchers and the participants. To minimize the power imbalance, they shared some aspects of their own immigrant identity and story with the participants so that the interview was a more reciprocal experience. Before commencing the interview they made the intent of the research very clear, discussed with them how their information will be used and stored. The researchers also conducted the interview in a location of the participant′s choice, where he or she would feel most comfortable answering questions.

Study data were collected from Calgary and Edmonton, the two largest cities in the province of Alberta, Canada. To participate in the study, an individual must be: (a) an immigrant worker 18 years or older from a linguistic or visible minority background, (b) a legal resident in Canada for less than 10 years, and (c) an immigrant who entered Canada through a formal immigration route. Undocumented workers were excluded from this study.

Participants were informed about the research by immigrant service providers operating in Calgary and Edmonton. Fifty interested individuals contacted the researchers, of which 42 (23 male, 19 female) agreed to participate in the interviews. Written informed consent was obtained from participants before they participated in the research interviews. Each participant was given a CAD 50 gift card in appreciation of the time he/she took to participate in the study. Interview data were collected from 2017 to 2019 by trained graduate research assistants through face-to-face interviews of approximately 90 min. Before the interview the participants were asked to complete a short sociodemographic survey. Data collection commenced after the receipt of ethics approval from the University of Calgary Conjoint Faculties Research Ethics Board (REB16-1675).

### 2.2. Data Analysis

Data collection and data analyses progressed simultaneously. Theoretical saturation was achieved following the analysis of 30 interviews; however, all the 42 interviews were analyzed. There were no females in the construction and oil and gas sectors. During the interviews, the researchers wrote memos of their impressions of important discussions and emerging issues. ATLAS.ti 7.0 software (ATLAS.ti Scientific Software Development GmbH, Berlin, Germany) was used to assist with the analysis. After an initial coding by a pair of graduate students with training in qualitative research, a coding framework was developed. All the coding was reviewed by the principal investigator. Biases arising from the positionality of the research team members in terms of professional belief, immigration status, social location, theoretical orientation, and emotional response to the participants were discussed during team meetings, as were the coding decisions and the memos written during the interviews. Using a constant comparative approach [36], the codes were grouped into themes to generalize the data and to identify key issues related to the research questions. A clear audit trail, accurate documentation of the research and analysis procedures, and hard copies of the transcriptions were maintained.

## 3. Results

### 3.1. Socio-Demographics of Participants

The research sample included 24 participants who had each experienced and reported a workplace injury/illness to their employer (injured participants), 15 participants who had not experienced an injury (non-injured participants), and 3 participants who had each experienced an injury but had not reported it. The three participants who did not report their injuries said they were unsure if their injury was serious enough to be reported. They blamed themselves for the injury and felt that reporting the injury might lead to them being judged as incompetent.

Participants originated from retail (33%), construction (19%), and oil and gas (14%) sectors (Table 1). Seventy-nine percent of the participants had bachelor, master, or doctoral degrees. The sample included lawyers (qualified overseas), tradesmen, engineers, business managers, and health-care professionals (doctors, nurses, social workers). Except for the social worker and the nurse who had managed to find work in their fields, participants were working as cooks, cleaners, sales or shop assistants, construction workers, warehouse workers, and machine assistants. The participant sample was culturally diverse and included Filipinos, Africans, South Asians, Chinese, Hispanics, and Eastern Europeans.

Participants had sustained work-related injuries of varying intensities. Examples of injuries included: injuries to the back, shoulder, or arm; repetitive strain injuries (RSI); and burns from chemicals and hot liquids. At least six participants had experienced workplace bullying or harassment. Some participants were able to link their injury to a mismatch between their educational qualifications and the type of work they had undertaken. One participant reported, “Back home, I was a public health specialist and I did a healthcare management job. And here I do physical labor, that is why I may get hurt, but I did not get hurt in my back home job because there I did not do any physical labor job like this”.

Only 10 participants (6 from the injured group and 4 from the non-injured group) were union members. The majority of them were from the construction and oil and gas sectors. The remaining 32 participants reported that they were unaware of the existence of a union in their company. Five participants who were union members said that they felt safer because of their unions. Two injured participants reported that they did not feel supported by their union in their time of need. The other three union members had not sought any help from their union at the time of these interviews. Our interviews suggested that most workers would have joined an appropriate union if they were aware of it.

Data analysis yielded three themes related to the research questions: (1) comprehension of OHS, (2) awareness of worker rights, (3) challenges of workplace safety risks and hazards.

### 3.2. Comprehension of OHS

A good understanding of OHS includes awareness of safety hazards in the workplace, how to perform a job safely, knowledge of worker rights, and responsibilities in the workplace, including the right to refuse dangerous work and the right to receive support or compensation in the event of injury [8,22]. All the study participants were aware of the importance of working safely and using personal protective equipment (PPE). When asked what they understood by OHS, their responses varied. Most participants described their experience of some aspects of OHS and left out others. Several participants said that OHS was about knowing the dangers in their work, following safety rules, knowing what to do in the event of an accident, using PPE if provided, and avoiding injury. All the participants were able to identify some of the dangers associated with their jobs. Participants reported that they had learned about these dangers through their coworkers/supervisors and their personal experience. Some participants had become aware of job-related hazards only after receiving an injury. One participant had been told by her supervisor to keep away from machines but was not informed of other dangers that did not involve machines: “actually before the incident I had no idea, only after the incident I was informed of some stuff, so, my understanding was that we need to be careful only when working with machines.”

There were no marked differences between injured and non-injured participants in their understanding of OHS, as highlighted by the following responses:

*OHS is knowing what to do in the event of a work-related accident, it is how to probably to keep yourself from getting hurt or injured or anything like that*.

(IN1)

*That is when the company will be providing you with information to ensure your own safety and also your colleagues′ safety in the work place … And then of course they provide you with information, as to what you are going to wear while doing your work*.

(II3) (injured participant)

Participants who sustained injuries and reported them to their supervisors and had accessed workers’ compensation were aware of injury reporting and the role of workers’ compensation in providing medical and financial support post injury.

Participants who worked in the oil and gas sector and in construction conveyed a more complete understanding of OHS than participants from retail, hospitality, and manufacturing sectors. The former workers were aware that it is the responsibility of the employer to provide training and to maintain and enforce safety standards; it is the responsibility of the employee to follow safety protocols and to refuse to undertake work in unsafe conditions. A participant whose work in the construction industry had been recently terminated after an injury offered the following testimony:

*It (OHS) is about your life, it is about your health and it is your responsibility to take care of that, you have to be careful! You cannot just say, “oh, let’s do it” (sign of rush work, careless work). So, you have to be calm down, relax and have to do things carefully, slowly, and safely. You cannot rush, nobody can push you. If you feel somebody is pushing you and if it can be dangerous for you, just stop*.

(II4)

The modified duties that the employer provided to this participant when he returned to work had aggravated his injury. The reasons for the more complete understanding of OHS presented by participants who worked in the oil and gas sector were: (a) they had undergone special OHS training provided by private safety training providers and had the requisite safety certificates for obtaining employment in these sectors, (b) these participants had worked in Canada for at least three years, and (c) some of these participants had worked in a similar industry overseas.

Whereas most participants associated OHS with personal physical safety, participants who had experienced mental distress due to racism or harassment at work took a more holistic view and included emotional safety as part of OHS. A participant who had experienced the trauma of racial discrimination explained:

*Safety, to me it’s more like mental, psychological safety, like others don’t abuse me verbally, that they don’t isolate me because I’m an immigrant, ignoring me and if you speak broken English and if you have an accent that you don’t have anything to offer*.

(IN10)

Overall, the findings suggest that the majority of participants had an incomplete understanding of OHS. Participants’ comprehension of OHS varied depending on their years of work experience in Canada, the industry they work in, the safety training they received, and their experience of injury.

#### 3.2.1. Acquiring OHS Knowledge

Some participants were familiar with the OHS concept because occupational health and safety was practiced to varying degrees in the workplaces in their countries of origin. Participants from India and Bangladesh said that OHS regulations exist in these countries but are not followed rigorously. One participant noted “here (Canada), too, it is not 100%”. The participant added that safety was often compromised in the pursuit of profit. Participants from Eastern Europe reported that OHS standards in their country were much higher than OHS standards in Canada and that Canadian regulations regarding the use of personal protective equipment (PPE) can be harmful in some instances. One participant said, “the safety glasses I must wear during welding turn sweaty and foggy very fast in summer and in winter. If I wear my glasses properly, I do not see anything. If I do not wear my safety glasses properly, I could be fired”. This worker had been unable to discuss this important safety issue with his Canadian supervisor because he was unsure of his supervisor′s reaction and he was also worried that reporting this could affect his job. Participants who received specialized training from safety training providers in Canada felt that their training was useful, although some participants felt that the safety training curriculum did not consider the interpersonal work environment. They felt the interpersonal relationships in the workplace among coworkers and between workers and supervisors can create stress if workers do not know how to handle them.

#### 3.2.2. Participants′ Perspectives of the OHS Training They Received at Their Workplaces

Workplace OHS training and on-the-job experience are key elements for acquiring OHS knowledge. The majority of participants acquired knowledge about OHS at their workplaces in Canada—either formally through OHS training conducted by their employers or informally from supervisors, coworkers, and their own work experiences. Large enterprises provided OHS information as part of their orientation programs for new employees.

Only 8 of the 42 participants expressed satisfaction with the OHS training they had received from their employers. All the other participants were dissatisfied with aspects of the safety training they received. Participants said that the training provided was too general; it lacked worksite and job-specific safety content and included no information about how to perform the job safely. One participant reported:

*During the orientation they gave us a document to study and a questionnaire to fill out, so, that was the basics of the safety training—general knowledge stuff. It was not a proper orientation to safety*.

(IN3)

Since workplace hazards are unique to the workplace, general OHS orientations did not provide enough information about specific workplace hazards. Participants felt a need for OHS training provided by workers experienced with workplace hazards and said that the handouts and questionnaires on OHS did not provide information on how to do the job safely. Several participants expressed a need for practical hands-on training provided by experienced workers. One of them said:

*You know, my workplace is a warehouse and it is a busy place and there are lots of machineries, and workers roam around these machineries. So definitely there are so many hazards. Giving us sheets/documents for reading and signing documents are not enough. Workers need more practical training provided by a group of professional or experienced persons, not just some documents for reading and keeping them*.

(IN8)

Some businesses posted OHS information in conspicuous places where it is likely to come to the attention of workers. While the law requires employers to do this [37], employers should ensure that new immigrant workers are given the time to read and understand this information. A participant who worked in one such business observed that workers like him did not have the time to read these postings during their busy work schedules. He suggested that spending time reading these postings could arouse the manager’s displeasure and suspicion:

*My manager might say “Oh you′re just standing there reading that stuff, why don′t you go back to your work?” I am scared I might get fired because of that*.

(IN6)

Several participants observed that their OHS orientation did not include information about accident reporting policies and post-injury supports, including workers’ compensation. Employers might suppress this information, fearing that workers might make false injury claims that could have an adverse impact on the premiums they pay to the Worker′s Compensation Board. As explained by one participant:

*They tell you to be careful, but they never tell you that there is something like WCB! They are very clever! But, yeah, they may tell you if you ask them after injury. They have first aid and they will provide you when you have your hands burn or some other minor injury…. And our employers do not tell us about that [WCB] because they have also to pay money or compensation for injured workers. Reporting an injury by a worker means financial loss for a company and so employers try not to tell their workers about those things*.

(IN9)

OHS training and support for new immigrants must be provided in common ethnic languages, in addition to English and French. Since this is not the case, participants with poor English language skills experienced difficulties understanding the training and feared that this would increase their risk of injury. An important issue that was missing in the OHS training was the recognition of bullying and harassment as work-related injuries. New immigrants who are not informed about harassment and bullying are likely to develop mental health issues when faced with these behaviors. A participant who developed anxiety and sleeplessness was taking antidepressant medication to cope with racial harassment from her team supervisor and co-workers. She explained:

*I was struggling for these ten weeks. My coworkers and team manager kept saying, “don′t do this, don′t do that! Don′t you know how to prepare a salad? You know, they often laughed at me for my illiteracy on preparing food. I said—you know, I don′t know how to prepare these kinds of food. I decided to quit and told my manager “I cannot deal with this anymore.” She moved me to another department*.

(II8)

Despite their dissatisfaction with the OHS training, participants did not ask for information or support from their supervisors and senior coworkers. Few participants were aware that Canadian employers expect workers to ask questions of their supervisors. People from some cultural backgrounds consider it disrespectful or confrontational to ask questions of their supervisors. Some participants were worried about their language barriers, while others were unsure of their supervisor′s reactions and feared reprisal. A participant recalled how her supervisor had reacted to her question about safety:

*Sometimes I don′t understand some Canadian terms. So, if the boss is talking to me, and I did not understand the term, I usually say, “come again” or “pardon me,” and she gets irritated…* *If you express yourself in the way they do, then there is going to be a good relationship [with your supervisor and colleagues] … But how can I even build a good relationship with them when I cannot even express myself in the way that they understand?*

(IN7)

While such reactions from supervisors reflect a lack of sensitivity to differences in language competency and accent, they also frame the participant′s communication difficulties as personal limitations. Such behaviors from supervisors not only block new immigrant workers from asking questions to increase their awareness of safety, they can also lead to workers blaming themselves for their poor communication skills.

Some participants received information about safety aspects of the equipment they used only after they or a coworker sustained an injury. A participant who had learned to operate a machine from a coworker sustained two serious injuries to his arm from the machine. He observed, “They don′t tell [you] at first everything. If something happens to you, that′s when they come to you and tell you” [about safety operations, injury reporting, workers compensation]. “They just tell me about their issue” [the work they want him to do for them], “but not about my safety.”

(II10)

When a newcomer lands in Canada, his/her priority is to get a job. Learning about occupational health and safety (OHS) and his/her rights are often not the priority for a newcomer because he/she does not expect to suffer a workplace injury. Therefore, a newcomer might not pay much attention to OHS information and might forget what he/she has learned. This happened to several participants. They reported that they had not paid sufficient attention to what was taught during orientation and blamed themselves for the injury they sustained. This highlights the importance of providing workers with periodic refresher courses on OHS.

### 3.3. Participants′ Awareness of Canadian Workers′ Rights and Entitlements

A good understanding of their rights and entitlements under OHS is critical for workers. The right to refuse unsafe work was the only right that several participants were informed about. They were aware that in the event of an accident, they should report it to their supervisor. Only a few participants discussed safety issues with their supervisors and participated in safety meetings in their workplaces. The majority of participants were unaware of other rights, such as the right to know about hazards in the workplace and the right to participate in hazard identification and remediation. Participant awareness of the right to refuse unsafe work, however, did not mean that participants were able to assert this right. Participants reported that they seldom refused unsafe work because they feared a loss of work hours and job termination. One participant explained:

*My coworkers, they got ill (after they performed unsafe work), and they can′t come to work the following day and they requested that they should go for a medical check up to make sure everything is all right… The company was not comfortable with the request… so they got sent home after that… I know some other guys as well that also experienced this illness but they fear “Oh maybe if I mention it, I might be sent home just like the other two guys.”… So, they try to manage the illness… instead of letting the employer know that “Oh this job is not safe.”… So, they try to protect the job over their health*.

(IN2)

Immigrant workers might also regularly accept unsafe work because of their disadvantaged financial situation; by doing so, they allow employers to take advantage of them. A participant who had seen several of her immigrant colleagues regularly taking on unsafe work called it “normalizing injustice.” She commented, “What they [these workers] are really doing is normalizing injustice and accepting exploitation … They′re normalizing it because they all have families here, they have mortgages on the house, so, they′re just accepting any type of abuse …” Some participants had undertaken unsafe work because they were unaware at the time that it was unsafe.

Participants, especially those who were very new (less than 3 years in Canada), were unaware of employment standards, such as their entitlement to disability leave, their right to take work breaks, and their right to be paid for overtime hours. Some had learned about overtime policies and taking breaks informally from coworkers. Regarding taking work breaks, a participant who worked for a large well known retail chain observed:

*I know the policy or labor law says that we should get a 30 min-break after 4–5 h of work, but we don′t get this practically. They don′t follow the regulation fully, I think! If we take like 30 min-break they don′t pay us. If we take a 15 min-break they pay*.

(IN4)

These findings suggest that even if workers learn about their rights and employment standards from coworkers or other sources, some employers/supervisors will use their power to revise these policies/standards to suit their business interests. It makes it easier for employers/supervisors to do this when workers are non-unionized, as was the case with the majority of study participants.

Although most participants were generally aware that they must report workplace injuries to their supervisor, very few were informed about injury reporting procedures and the role of the Workers Compensation Board (WCB) before their injury occurred. Several participants were unaware of the possibility of returning to work on modified duties. This information is very important for new immigrants who are at a higher risk than native-born workers of sustaining injury. A participant noted:

*I did not really know about WCB, the procedures of reporting injury, or any worker′s rights. I think it would be great to organize some talks for new immigrants so as to let them know what is workplace related injuries. I think what is most important is that you are knowledgeable about the procedures of reporting an injury. New immigrants usually start their life here with labor jobs, not office work, so they have higher chance of getting injured*.

(II9)

### 3.4. Challenges of Facing Workplace Risks and Hazards

All the study participants were able to identify at least some risks and hazards associated with their jobs and worksites. They reported that they had learned these hazards through experience, through interactions with supervisors/coworkers, and in some cases, after being injured. Although participants provided several examples of hazardous and unsafe work conditions they were exposed to—exposure to toxic fumes that made them feel sick, having to stand for eight hours at a stretch, heavy lifting, and working with faulty equipment—they did not speak up because of fears of being terminated by their employer.

Participants faced another set of challenges when their employers/supervisors failed to enforce safety standards. Only eight participants noted that their employers enforced compliance with OHS regulations and suspended or terminated workers who did not observe the OHS rules. Several participants said that their employers were lax about enforcing safety and this allowed some workers to flout safety rules, even when this was hazardous for other workers. As observed by a participant:

*These workers put away their safety guards [PPE] by themselves. Sometimes I do not understand these Canadian people. They know safety rules, but they do not follow them and the manager does not say anything*.

(II15)

Non-compliance with OHS is associated with an increased risk of injury, disability, and even death [37]. The finding that many employers were able to get away with not enforcing safety compliance suggests a failure on the part of government legislation to make employers accountable for a safety culture.

While these challenges were experienced by all workers engaged in precarious work, several participants experienced feelings of disempowerment because of lack of support and the differential treatment they experienced from some of their supervisors and coworkers. Participants observed that immigrant workers like themselves were treated less favorably than Canadian-born workers by workplace supervisors. Some participants reported being pressured by supervisors or senior coworkers to take on additional duties, something that their Canadian-born peers were seldom asked to do. They stated that immigrant workers frequently took on “additional duties and performed workloads equivalent to two or three people in an 8-h shift.” Sometimes, participants took on an additional workload to gain the approval of or acknowledgement from a supervisor. As explained by one participant:

*Immigrant workers are seen as less capable than the Canadian-born workers. So, by their work they have to prove that they are also capable of doing all types of work equally, and sometimes more efficiently, than the Canadian-born workers*.

(II6)

Participants said they were often asked to take on work that their Canadian peers did not want. Some participants noted that as new immigrants, they could not afford to make mistakes, as their work was more closely monitored by supervisors and they could be terminated. One of these participants explained:

*If you are an immigrant worker, and if you make any mistake, there is more possibility you will be noticed by the employer. But if a Canadian-born worker makes a similar mistake, he will not be brought under scrutiny. A Canadian-born worker′s mistake or fault will be overlooked. As an immigrant worker, I have to follow every rule and regulation of the workplace. I can be can be fired any time if I make a mistake*.

(IN11)

Some participants felt they were treated differently from Canadian workers by supervisors and coworkers because they were from poor countries.

*They don’t care about your educational qualification and experience or individual ability. There are instances where a worker comes from a poor country and has higher level of educational qualification, but another worker comes from a rich country and has no higher educational qualification and is getting better treatment in workplace*.

(II12)

When participants tried to assert themselves and requested better working conditions, their requests were ignored or they were asked to leave. Some highly skilled professionals narrated their experiences of feeling devalued and disrespected in their workplaces.

*I can say that I experienced humiliation in Canada. I am a specialist, but I am told to do jobs that do not require any qualification. At my job, only Canadian welders have rights… I felt like I was garbage who came from (country of origin). We got very good education in (country of origin). We taught Canadian journeymen how to weld properly. However, here they work as welders, but we are their helpers… there is a huge difference how the supervisors treat Canadians and us. Canadians can take a break any time and go to washroom or somewhere else, go to smoke. We were not allowed to stop work*.

(IN15)

Not all participants, however, felt unsupported by their supervisors. Some participants shared that they had a good relationship with their supervisors and their workplaces were culturally friendly.

## 4. Discussion

This study presents exploratory data on new immigrant workers′ perspectives of occupational health and safety (OHS) in Alberta, Canada, and work conditions that lead to or have the potential to cause a workplace injury. The findings are based upon the testimonies of a group of well-educated visible and linguistic minority workers who entered Canada as legal immigrants less than ten years ago. The findings complement and build on previous research [4,6,10,14,16,21,22,38]. This research makes important contributions to theory, practice, and policy.

### 4.1. Theoretical Connections

The findings contribute to the OHS vulnerability framework [22] by highlighting personal and contextual factors that can increase the risk of injury for new immigrant workers. The findings that many participants with postsecondary and professional qualifications were employed in jobs for which they have little training, and the fact that some highly qualified participants linked their injury/illness to a mismatch between their educational qualifications and the low-skilled work they had undertaken, both complement previous research [28,39]. The findings support the person–environment fit theory [40], which posits that incongruence between a worker’s desires, interests, and values and the work that he/she is engaged in can lead to poor mental and physical health. A recent 14-year follow-up study [41] uncovered an elevated risk of diabetes mellitus in workers who are overqualified for their jobs. Thus, it is important to prepare highly qualified immigrant workers to access appropriate training for the work they have undertaken to minimize the risks to their health. At a broader level, the findings contribute to the theoretical discourse around ethnic and racialized immigrant workers from the developing world who are still locked in forms of labor exploitation that marked the spread of global capitalism in the latter decades of the twentieth century [6]. The current surplus of under- and unemployed immigrant workers in the labor market, as a result of the crisis in global capitalism, not only places a downward pressure on their wages but allows employers to extract their labor power [10] with little regard for their occupational and cultural safety. The findings show how employers take advantage of the immigrant worker’s disadvantaged social and financial status despite the spread of neoliberal policies that proclaim opportunity and fairness are available to all [6].

Previous research suggests that new immigrant workers have a limited understanding of OHS and, therefore, a higher risk of injury than Canadian-born workers [15,16]. Current findings, while broadly consistent with previous research, suggest that new immigrant workers’ understanding of OHS varies and depends on their years of work experience in Canada and overseas, the industry in which they have previously worked, the safety training they have experienced, and their encounters with injury. Due to a disparity between OHS regulations and practices, the relatively high education background of the immigrants in this study did not help much with their adaptation to and uptake of the occupational safety requirements. New immigrant workers have a varied understanding of OHS and this must be considered when providing them with OHS training and support.

The testimony of new immigrant workers presented here highlights the challenges participants faced in acquiring OHS knowledge and in practicing work safety. Since most new immigrants depend on their workplaces to acquire OHS knowledge, a major challenge arises when workplaces do not provide the training and support needed to practice work safety. Although OHS legislation in Alberta is designed to ensure that employers provide workers with training, supervision, and the resources needed to do their jobs in a safe manner [31], the findings suggest that many employers in Alberta are failing to meet these obligations. While some employers did not provide any OHS training, the OHS training that others provided often did not meet the specific needs of the participants in this study. These findings agree with conclusions reached in other research that found a low level of compliance with basic OHS obligations in Alberta′s employers [42].

Poor quality and inadequate OHS training poses a safety risk for all workers, but this risk is higher for new immigrant workers. Several study participants, despite being highly qualified for their positions in industry, were not informed about issues that were critical for their safety, such as Canadian employment standards, injury reporting procedures, and post-injury support systems, including workers’ compensation. When employers and supervisors do not inform workers or delay informing workers about their rights to medical support in the event of injury because of fears that workers might make false WCB claims or take time off, they run the risk that workers will return to work partially recovered and sustain reinjury [20]. Participants′ experiences of being devalued and being treated as “others” are not surprising, given the high prevalence of racism and discrimination in Canadian workplaces [43]. According to Statistics Canada General Social Survey (2016), the presence of racism and discrimination in Canadian workplaces makes bullying and harassment of new immigrants inevitable [44]. Such experiences of discrimination, harassment and bullying, and feelings of disempowerment can lead to mental health problems among new immigrant workers and increase their vulnerability to work-related injury/illness [45].

### 4.2. Recommendations for Practice and Policy

The findings reveal that the OHS training and support participants received and the methods of delivering OHS information were not culturally friendly, did not include interpreters, and participants were often not provided with the requisite time and support to read and understand the OHS materials provided. In view of the inadequate OHS training and support new immigrants receive in industry, there is a growing recognition of the importance of providing OHS information to immigrant employees before they enter the workforce. Accordingly, OHS training programs and tool kits are being developed to prepare new immigrant workers for the workforce [17]. To be effective, these tool kits should be developed in consultation with immigrant workers who can provide first-hand information on the OHS training they need. Our interviews with study participants suggest that OHS training should include information on the following: workers′ rights and responsibilities, employment standards, information on harassment and bullying, post-injury supports, and the employer′s responsibility to provide OHS training and support to new employees. Since new immigrant workers experience challenges with communication and may be unaware of Canadian workplace norms and employer′s expectations, OHS training should include information regarding these aspects. Specifically, communication and relationship building with supervisors and coworkers should be included in this training. Immigrant-serving agencies can incorporate these resources into their settlement and employment preparation programs for newcomers.

An important finding of this research is that programs that aim to increase new immigrant workers′ awareness of OHS and of their rights and responsibilities are not enough to empower them to refuse unsafe work or to report their OHS concerns. For example, many study participants were aware of their right to refuse unsafe work but did not assert it because of fears of reprisal and termination, a finding that is in keeping with previous research [46]. Current findings also suggest that the negative attitude of some employers/supervisors toward new immigrant workers and their tendency to take advantage of the workers′ marginalized financial and social situations by asking them to perform additional work and unsafe work, have the potential to increase OHS risks for these workers. Given that this is the case even for highly educated immigrants in this study, it would not be hard to understand the additional stressors and challenges faced by immigrants with less favorable educational backgrounds and socio-economic status, or by those without legal status. It is important to note that the international crises emerging in different parts of the world will increase the challenges faced by future refugees seeking asylum in Canada.

The findings of this study call for government interventions that enforce the creation of safe workplace environments where new immigrant employees feel included and valued and can learn safe work practices. Since workplaces in Canada are becoming increasingly multicultural, the promotion of safe workplace environments for immigrants can be a good business investment for employers, as it will reduce accidents, injuries, and WCB claims [47,48]. Although the responsibility for promoting such environments starts with the management in large companies and with business owners in small- to medium-sized firms, the government has an important role to play in ensuring that employers create such environments. Research shows that employers need laws and regulations as incentives to implement such interventions [48]. In the following section, we outline what the government, service providers, and employers can do to create safe working environments for new immigrant workers.

In the current Alberta OHS system, penalties, such as fines and jail terms, can be levied for employers convicted of offences under the Occupational Health and Safety Act [34]. These penalties should be supplemented by incentives that make employers accountable for creating a safe workplace environment for all workers and, specifically, for new immigrant workers because of their high risk of sustaining workplace injury. Such incentives would include compulsory regular worksite inspections by OHS officers to ensure that hazards are recognized early [49] and mandatory OHS training for business managers, staff with supervisory responsibilities, small business owners, and new immigrant business owners to increase their awareness of and commitment to implementing a work safety culture. OHS training should include content that addresses biases about immigrant workers′ culture, language, and skills. Economic incentives, such as tax deductions, should be available for employers who prioritize safety. Additional incentives, such as bonuses, can be provided to business owners who make a commitment to safety, so that keeping the workplace safe is perceived to be a reward instead of a burden. Employers must be legally responsible for organizing job and worksite-specific safety training for all new workers and this training must be provided by competent staff. Culturally appropriate modes of delivery, including the use of pictograms, illustrations, and hands-on exercises that transcend cultural, educational, and linguistic differences, should be included in this training [50].

As language barriers, poor understanding of workplace culture and communication styles often cause immigrant workers to avoid seeking help from coworkers or supervisors; employers can appoint mentors who can provide timely onsite advice and help. Additionally, the government should implement a policy that makes comprehensive OHS training a legal requirement for all workers before or soon after they are employed. As discussed earlier, immigrant service providers can be involved in providing OHS training to newcomers and they should receive provincial funding for this purpose. In recent years, several prominent scholars in the field of OHS have supported the development of integrated health approaches that alter workplace policies and provide programs and practices that will protect workers from work-related health hazards. While empirical evidence supporting the effectiveness of these approaches is still emerging [51,52,53], such programs will benefit new immigrants, particularly because of their high risk of injury/illness and the frequent mismatch between their educational qualifications and the low-skilled jobs that many immigrants undertake.

### 4.3. Limitations of the Study and Relevance beyond Alberta

This study has a few limitations. The sample drawn from two localized geographic sites is small in size. The study participants were identified through immigrant-serving organizations and employment agencies, and their experiences may differ from immigrants who did not access these services. This study does not include the perspectives of employers and service providers on the difficulties experienced by new immigrant workers and the broader supports needed to improve their labor market productivity. Additionally, the study focused exclusively on legal immigrants who came to Canada in the last ten years. It did not examine the challenges experienced by undocumented migrant workers. A future study should focus on this population using critical ethnography as a research approach [54]. Despite these limitations, the study has strengths and the findings are useful beyond the Canadian context. Since about two-thirds of the participants reported having a university degree or even higher education, the study sample is weighted with respect to well-educated workers [55]. Few studies have examined the working contexts of educated and professional immigrants engaged in high-risk jobs and the challenges they experience in acquiring OHS information. The challenges that new immigrants currently face across the developed world occur in the context of government reforms and employer pressures to reduce costs associated with workplace injury, disability, and rehabilitation. The potential policy and practice recommendations that have been suggested to increase OHS knowledge among new immigrants and to promote a culture of workplace safety can reduce workplace injury, increase worker productivity, and can be applied across several jurisdictions in Canada and beyond.

## 5. Conclusions

This qualitative study provides important information about the limitations of current workplace OHS training and support provided by employers to new immigrant workers in Canada. Immigrant workers bring relatively high educational qualifications to Canada. The personal accounts of the challenges these immigrant workers face in acquiring occupational health and safety information, in practicing work safety, their experiences of being asked to take on hazardous work more often than local workers, and their inability to refuse such work, suggest that workplace environments play an important role in increasing new immigrant workers′ risk of workplace injuries. Given the educational capacity of immigrant workers, it is often a matter of providing the necessary resources they need in a culturally sensitive manner and rendering appropriate safety monitoring and enforcement. The findings suggest that governments, employers, and immigrant service providers should work in partnership to ensure that new immigrants acquire OHS knowledge and be apprised of their rights before or soon after they enter the workforce. The findings suggest a need for policy and legislation changes that will increase employers’ commitment and accountability to create safe and inclusive workplace environments for new immigrant workers.

## Figures and Tables

**Table 1:** Socio-demographics of participants.

Attributes	Injured	Non Injured	Injured but Did Not Report
***Gender***			
Male	12	10	1
Female	12	5	2
***Age***			
18–25	1		
26–35	6	2	
36–45	8	5	2
46–55	9	8	1
***Education***			
High school	5	3	
Bachelor′s degree	10	4	2
Master′s degree	8	7	1
PhD/MD	1		
Overseas trade certificate		1	
***Number of Years in Canada***			
1–2 years	3	4	
3–5 years	12	6	2
6 ≤ 10 years	9	5	1
***Region of Origin***			
Asia	11	5	2
Africa	5	6	
Eastern Europe	8	1	1
Central South America		3	
***Industry Employed***			
Construction	5	2	1
Healthcare	3	1	
Hospitality	3	2	1
Manufacturing	2	1	
Retail	6	7	1
Oil and Gas	5	1	
IT/other		1 (IT)	

## Data Availability

Data supporting the reported results are available on request from the corresponding author. The data are not publicly available due to the need to protect the privacy of the research participants.
